# Involvement of maternal embryonic leucine zipper kinase (MELK) in mammary carcinogenesis through interaction with Bcl-G, a pro-apoptotic member of the Bcl-2 family

**DOI:** 10.1186/bcr1650

**Published:** 2007-02-06

**Authors:** Meng-Lay Lin, Jae-Hyun Park, Toshihiko Nishidate, Yusuke Nakamura, Toyomasa Katagiri

**Affiliations:** 1 Laboratory of Molecular Medicine, Human Genome Center, Institute of Medical Science, The University of Tokyo, Tokyo, Japan

## Abstract

**Introduction:**

Cancer therapies directed at specific molecular targets in signaling pathways of cancer cells, such as tamoxifen, aromatase inhibitors and trastuzumab, have proven useful for treatment of advanced breast cancers. However, increased risk of endometrial cancer with long-term tamoxifen administration and of bone fracture due to osteoporosis in postmenopausal women undergoing aromatase inhibitor therapy are recognized side effects. These side effects as well as drug resistance make it necessary to search for novel molecular targets for drugs on the basis of well-characterized mechanisms of action.

**Methods:**

Using accurate genome-wide expression profiles of breast cancers, we found *maternal embryonic leucine-zipper kinase *(*MELK*) to be significantly overexpressed in the great majority of breast cancer cells. To assess whether *MELK *has a role in mammary carcinogenesis, we knocked down the expression of endogenous *MELK *in breast cancer cell lines using mammalian vector-based RNA interference. Furthermore, we identified a long isoform of Bcl-G (Bcl-G_L_), a pro-apoptotic member of the Bcl-2 family, as a possible substrate for MELK by pull-down assay with recombinant wild-type and kinase-dead MELK. Finally, we performed TUNEL assays and FACS analysis, measuring proportions of apoptotic cells, to investigate whether MELK is involved in the apoptosis cascade through the Bcl-G_L_-related pathway.

**Results:**

Northern blot analyses on multiple human tissues and cancer cell lines demonstrated that *MELK *was overexpressed at a significantly high level in a great majority of breast cancers and cell lines, but was not expressed in normal vital organs (heart, liver, lung and kidney). Suppression of *MELK *expression by small interfering RNA significantly inhibited growth of human breast cancer cells. We also found that MELK physically interacted with Bcl-G_L _through its amino-terminal region. Immunocomplex kinase assay showed that Bcl-G_L _was specifically phosphorylated by MELK *in vitro*. TUNEL assays and FACS analysis revealed that overexpression of wild-type MELK suppressed Bcl-G_L_-induced apoptosis, while that of D150A-MELK did not.

**Conclusion:**

Our findings suggest that the kinase activity of MELK is likely to affect mammary carcinogenesis through inhibition of the pro-apoptotic function of Bcl-G_L_. The kinase activity of MELK could be a promising molecular target for development of therapy for patients with breast cancers.

## Introduction

Breast cancer is one of the leading causes of cancer death in women worldwide. According to a 2002 estimate, more than 1,100,000 patients were newly diagnosed with breast cancer, and approximately 410,000 patients died of the disease [1]. Recent improvements in detecting breast cancer at an early stage through mammographic screening have contributed to a decrease in breast cancer-associated mortality. Mastectomy is among the first options for treatment of localized breast cancer. Despite surgical removal of primary tumors, however, relapse at local or distant sites occurs in a subset of patients, probably due to undetectable micrometastases at the time of diagnosis [2,3].

Cancer therapies directed at specific molecular targets in signaling pathways of cancer cells, such as tamoxifen, aromatase inhibitors and trastuzumab (Herceptin), have been proven to be useful for treatment of advanced breast cancer [4]. Tamoxifen and aromatase inhibitors suppress the estrogen-related signaling pathway and trastuzumab is the first approved monoclonal antibody for blocking the human epidermal growth factor 2 (HER-2/ErbB-2) signaling pathway [4,5]. Patients with tumors that express estrogen or HER-2 receptors can benefit from either of these therapies and are expected to have a better quality of life and prognosis. However, increased risk of endometrial cancer with long-term tamoxifen administration and of bone fracture due to osteoporosis in postmenopausal women undergoing aromatase inhibitor therapy are recognized side effects [6,7]. Due to the emergence of these side effects and also because of drug resistance, it is necessary to search for novel molecular targets for drugs on the basis of well-characterized mechanisms of action.

Toward the goal of identifying good molecular targets for drug development, we analyzed the detailed expression profiles of 81 breast tumors, representing 23,040 genes, using a combination of laser-microbeam microdissection and cDNA microarray analysis [8]. After comparing the expression profiles of these breast cancers with those of various normal human tissues [9], we focused on a gene termed *maternal embryonic leucine zipper kinase *(*MELK*) that was significantly overexpressed in the great majority of breast cancer cases examined.

In this study, we report evidence indicating that MELK functions as a cancer-specific protein kinase, and that down-regulation of *MELK *results in growth suppression of breast cancer cells. In addition, we demonstrate that MELK physically interacts and phosphorylates a long isoform of Bcl-G (Bcl-G_L_), a pro-apoptotic member of the Bcl-2 family. Our findings suggest that the kinase activity of MELK is likely to affect mammary carcinogenesis through inhibition of the pro-apoptotic function of Bcl-G_L_. We propose that MELK kinase activity could be a promising molecular target for the treatment of breast cancer.

## Materials and methods

### Cancer cell lines and clinical samples

Human breast cancer cell lines HBL100, HCC1937, MCF-7, MDA-MB-231, MDA-MB-435S, SKBR3, T47D, YMB1, BSY-1 and BT-20, human cervical adenocarcinoma cell line, HeLa, and monkey kidney cells transformed with SV40 T-antigen, COS7 cells, were purchased from the American Type Culture Collection (ATCC, Rockville, MD, USA). Human mammary epithelial cells (HMECs) were purchased from Cambrex Bio Science Walkersville, Inc. (Walkersville, MD, USA). HBC4 and HBC5 cells lines were kind gifts from Dr Yamori of the Division of Molecular Pharmacology, Cancer Chemotherapy Center, Japanese Foundation for Cancer Research. All cells were cultured under their respective depositor's recommendations: RPMI-1640 (Sigma-Aldrich, St Louis, MO, USA) for HBC4, HBC5, HCC1937, SKBR3, T47D, YMB1 and BSY-1 (with 2 mM L-glutamine); Dulbecco's modified Eagle's medium (Invitrogen, Carlsbad, CA, USA) for HBL100, BT-20 and COS7; EMEM (Sigma-Aldrich) with 0.1 mM essential amino acid (Roche, Basel, Switzerland), 1 mM sodium pyruvate (Roche), 0.01 mg/ml insulin (Sigma-Aldrich) for MCF-7; EMEM (Sigma-Aldrich) for HeLa; L-15 (Roche) for MDA-MB-231 and MDA-MB-435S; MEGM (Cambrex Bio Science) for HMECs. Each medium was supplemented with 10% fetal bovine serum (Cansera International, Ontario, Canada) and 1% antibiotic/antimycotic solution (Sigma-Aldrich). MDA-MB-231 and MDA-MB-435S cells were maintained at 37°C in humidified air without CO_2_. Other cell lines were maintained at 37°C in humidified air with 5% CO_2_. Tissue samples from surgically resected breast cancers, and their corresponding clinical information, were obtained from the Department of Breast Surgery, Cancer Institute Hospital, Tokyo, after obtaining written informed consent.

### Semi-quantitative RT-PCR analysis

Total RNAs were extracted from each microdissected breast cancer clinical sample and from microdissected normal ductal cells, and polyA(+) RNAs were isolated from normal mammary gland, lung, heart, liver, and kidney (Takara Clontech, Kyoto, Japan). Subsequently, T7-based amplification and reverse transcription were carried out as described previously [10]. We prepared appropriate dilutions of each single-stranded cDNA for subsequent PCR using the *glyceraldehyde-3-phosphate dehydrogenase *(*GAPDH*) gene as a quantitative control. The PCR primer sequences were: 5'-GCTGCAAGGTATAATTGATGGA-3' and 5'-CAGTAACATAATGACAGATGGGC-3' for *MELK*; 5'-CGACCACTTTGTCAAGCTCA-3' and 5'-GGTTGAGCACAGGGTACTTTATT-3' for *GAPDH*.

### Northern blot analysis

Total RNAs were extracted from breast cancer cell lines using the RNeasy kit (Qiagen, Valencia, CA, USA) according to the manufacturer's instructions. After treatment with DNase I (Nippon Gene, Osaka, Japan), mRNA was isolated with a mRNA purification kit (GE Healthcare, Buckinghamshire, United Kingdom) following the manufacturer's instructions. One microgram of each mRNA, along with polyA(+) RNAs isolated from normal mammary gland, lung, heart, liver, kidney and brain (Takara Clontech), were separated on 1% denaturing agarose gels and transferred to nylon membranes (breast cancer Northern blots). Breast cancer Northern blots and human multiple-tissue Northern blots (Takara Clontech) were hybridized with [α^32^P]-dCTP-labeled PCR products of *MELK *cDNA prepared by RT-PCR (see below). Pre-hybridization, hybridization and washing were performed according to the supplier's recommendations. The blots were autoradiographed with intensifying screens at -80°C for 12 days. A probe cDNA (542 base-pairs (bp)) for the sequence in the 3' untranslated region of *MELK *cDNA (GenBank accession number NM_014791) was prepared by PCR using the primer set 5'-TTATCACTGTGCTCACCAGGAG-3' and 5'-CAGTAACATAATGACAG ATGGGC-3' and were radioactively labeled using the megaprime DNA labeling system (GE Healthcare).

### cDNA library screening

We constructed a cDNA library using polyA(+) RNA obtained from T47D breast cancer cells and the Superscript™ plasmid system coupled with Gateway™ technology for cDNA synthesis and a cloning kit (Invitrogen). We screened 3 × 10^6 ^independent clones from this library with a cDNA probe corresponding to nucleotides 1,251 to 2,094 (843 bp) of the *MELK *cDNA sequence (GenBank accession number NM_014791).

### *In vitro *translation assay

Three variants of *MELK *(V1, V2 and V3) that were cloned into the pSPORT-1 expression vector were used as templates for transcription/translation experiments *in vitro*. The plasmids (1 μg) were transcribed and translated using a TNT Coupled Reticulocyte Lysate system (Promega, Madison, WI, USA) in the presence of ε-labeled biotinylated lysine-tRNA according to the manufacturer's instructions. Proteins were electrophoresed by SDS-PAGE with a 5% to 20% gradient. After electroblotting, the biotinylated proteins were visualized by binding streptavidin-horseradish peroxidase followed by chemiluminescent detection (GE Healthcare).

### Plasmid construction

To construct *MELK *expression vectors, the entire coding sequences were amplified by PCR using KOD-Plus DNA polymerase (Toyobo, Osaka, Japan) and cloned into the *Eco*RI and *Xho*I sites of the pCAGGSnHC expression vector in frame with the HA (hemagglutinin)-tag at the carboxyl terminus. The primer set used for PCR reactions with wild-type MELK-V1 was: forward, 5'-CGGAATTCACTATGAAAGATTATG ATGAAC-3' (underlining indicates the *Eco*RI site); reverse, 5'-AAACTCGAGTACCTTGCAGCTAGATAGGAT-3' (underlining indicates the *Xho*I site). Kinase-dead mutant MELK (D150A) was generated with a QuikcChange Site-Directed Mutagenesis kit (Stratagene, La Jolla, CA, USA) using the following primers; forward, 5'-CATAAATTAAAGCTGATTGCCTTTGGTCTCTGTGCAAAACC-3'; reverse: 5'-GGTTTTGCACAGAGACCAAAGGCAATCAGCTTTAATTTATG-3'.

For construction of the *Bcl-G*_*L *_expression vectors, the corresponding open reading frame was generated by RT-PCR using human testis mRNA as a template and the following set of primers (underlining of forward and reverse primers indicates *Not*I and *Xho*I sites, respectively): forward, 5'-ATAAGAATGCGGCCGCGATGTGTAGCACCAGTGG-3' for full-length *Bcl-G*_*L *_(FL); reverse, 5'-CCGCTCGAGTTACTATCAGTCTACTTCTTCATGTG-3' for full-length *Bcl-G*_*L *_(FL); forward 5'-ATAAGAATGCGGCCGCGATCCCCCTAGATGATG-3' for N-1; forward 5'-ATAAGAATGCGGCCGCGTGGCCTTGCAGAAATTC-3' for N-2; forward 5'-ATAAGAATGCGGCCGCGGAATACCAAGATTCGC-3' for N-3; forward 5'-ATAAGAATGCGGCCGCGCAGGCAGGAGGCTTC-3' for N-4; reverse 5'-CCGCTCGAGTTACTATCAGAAGTTCTCTTTCAGGT-3' for C-1; reverse 5'-CCGCTCGAGTTACTATCATCTGGGGTCCACACCCA-3' for C-2; reverse 5'-CCGCTCGAGTTACTATCACAGCTCAACAATTTTGG-3' for C-3. The reverse primer of the FL construct was commonly used for N-1, N-2, N-3 and N-4 constructs, and the forward primer of it was commonly used for C-1, C-2 and C-3 constructs. These amplified DNAs were cloned into *Not*I and *Xho*I sites of the pCAGGSn3FH vector in frame with Flag-tag at the amino-terminal site. DNA sequences of all constructs were confirmed by DNA sequencing (ABI3700, PE Applied Biosystems, Foster, CA, USA).

### Gene-knockdown of *MELK *by siRNA

We established a vector-based RNA interference (RNAi) expression system using a psiH1BX3.0 small interfering (si)RNA expression vector as described previously [11]. siRNA expression vectors against *MELK *(psiH1BX-*MELK*) and scramble control (SC; the gene coding for 5S and 16S rRNAs in the chloroplast of *Euglena gracilis*; psiH1BX-SC) were prepared by cloning of double-stranded oligonucleotides into the *Bbs*I site of the psiH1BX3.0 vector. The target sequence of synthetic oligonucleotides for siRNA were: si-#1, 5'-TCCCACTTGCCTGCCATATCCTTTTCAAGAGAAAGGATATGGCAGGCAAGT-3' and 5'-AAAAACTTGCCTGCCATATCCTTTCTCTTGAAAAGGATATGGCAGGCAAGT-3', si-#2, 5'-TCCCCTATCCTGTTGAGTGGCAATTCAAGAGATTGCCACTCAACAGGATAG-3' and 5'-AAAACTATCCTGTTGAGTGGCAATCTCTTGAATTGCCACTCAACAGGATAG-3', si-#3, 5'-TCCCGACATCCTATCTAGCTGCATTCAAGAGATGCAGCTAGATAGGATGTC-3' and 5'-AAAAGACATCCTATCTAGCTGCATCTCTTGAATGCAGCTAGATAGGATGTC-3', si-#4, 5'-TCCCAGTTCATTGGAACTACCAATTCAAGAGATTGGTAGTTCCAATGAACT-3' and 5'-AAAAAGTTCATTGGAACTACCAATCTCTTGAATTGGTAGTTCCAATGAACT-3'. SC, 5'-TCCCGCGCGCTTTGTAGGATTCGTTCAAGAGACGAATCCTACAAAGCGCGC-3' and 5'-AAAAGCGCGCTTTGTAGGATTCGTCTCTTGAACGAATCCTACAAAGCGCGC-3'. Underlining indicates siRNA-targeting sequences designed from *MELK *mRNA (GenBank accession number NM_014791). All constructs were also confirmed by DNA sequencing.

Human breast cancer cells lines T47D and MCF-7 were plated in 15 cm dishes (4 × 10^6 ^cells/dish), and transfected with 16 μg of psiH1BX-*MELK *(si-#1, si-#2, si-#3 and si-#4) and psiH1BX-SC (SC; negative control) siRNA plasmids using the FuGENE6 transfection reagent (Roche) according to the supplier's recommendations. At 24 hours after transfection, cells were re-seeded for the following experiments at the densities given: 1 × 10^6 ^cells/10 cm dish for semi-quantitative RT-PCR and western blot analyses; 3 × 10^6 ^cells/10 cm dish for colony formation assay and 5 × 10^5 ^cells/well for MTT (3-(4,5-dimethylthiazol-2-yl)-2,5-diphenyltetrazolium bromide) assay. We selected T47D or MCF7 cells transfected with *MELK*-siRNAs in medium containing 0.7 mg/ml or 0.6 g/ml neomycin (geneticine; Invitrogen), respectively. Four days after neomycin selection, 0.5 μg of total RNA extracted from these cells was reverse-transcribed into cDNA using Superscript II (Invitrogen) to examine the knockdown effect of siRNAs by semi-quantitative RT-PCR using specific primer sets for *MELK *and β*2MG *(encoding beta-2-microglobulin). Cells were also harvested four days after neomycin selection for western blot analysis using anti-MELK antibody (Cell signaling technology, Boston, MA, USA). The specific primer sets for RT-PCR were: 5'-TTATCACTGTGCTCACCAGGAG-3' and 5'-CAGTAACATAAT GACAGATGGGC-3' for *MELK*; 5'-TTAGCTGTGCTCGCGCTACT-3' and 5'-TCACATGGTTCACACGGCAG-3' for β*2MG *as a quantitative control. Transfectants expressing siRNA were grown for 3 weeks in selective media containing neomycin, then fixed with 4% paraformaldehyde for 15 minutes before staining with Giemsa's solution (Merck, Whitehouse Station, NJ, USA) to assess colony number. To quantify cell viability, MTT assays were performed at seven days after selection with Cell Counting Kit-8 (Wako, Osaka, Japan) according to the manufacture's protocols. Absorbance at 570 nm was measured with a Microplate Reader 550 (Bio-Rad, Hercules, CA, USA). These experiments were performed in triplicate.

### Generation and purification of His-tagged recombinant MELK

The entire coding sequences of wild-type MELK (WT-MELK) and kinase-dead MELK (D150A-MELK; asparate changed to alanine at the 150th residue in the kinase subdomain VII, which is considered as the ATP binding site [12]) were subcloned into the pET21a vector (Merck Novagen, Darmstadt, Germany). The WT-MELK and D150A-MELK recombinant proteins were expressed in *Escherichia coli *strain BL21 codon-plus (DE3) RIL competent cells (Stratagene), and cultured in TBG-M9 medium (1% tryptone, 0.5% NaCL, 0.5% glucose, 1 mM MgSO_4_, 0.1% NH_4_CI, 0.3% KH_2_PO_4_, 0.6% Na_2_HPO_4_). After induction with 0.5 μM isopropyl-b-D-thiogalactopyranoside (IPTG) at 25°C for 5 hours, the bacteria pellet was suspended with lysis buffer, and was purified with Ni-NTA superflow (Qiagen) under nondenaturing conditions according to the supplier's instructions. The entire coding sequence of BCL-G_L _was also subcloned into the pGEX-6P-1 vector (GE Healthcare). The glutathione S-transferase (GST)-Bcl-G_L _recombinant protein was expressed in *Escherichia coli *strain BL21 codon-plus RIL competent cells (Stratagene). Purification of the recombinant proteins was performed using Glutathione Sepharose 4B beads (GE Healthcare) under nondenaturing conditions according to the supplier's instructions. For confirmation of direct binding of BCL-G_L _and MELK, we removed GST from GST-fused BCL-G_L _protein using PreScission protease (GE Healthcare) according to the supplier's instructions.

### Protein pull down assay

HBC4 cells were lysed with lysis buffer (40 mM Tris-HCL (pH 7.5), 1% Trition X-100, 2.5 mM EDTA, 15 mM dithiothreitol (DTT)) including 0.1% protease inhibitor cocktail III (Calbiochem, San Diego, CA, USA) as previously described [13] with a minor modification. We incubated 7.5 mg of cell lysates with 0.5 μM of WT-MELK or D150A-MELK recombinant proteins pre-immobilized on Ni-NTA agarose beads in reaction buffer containing 50 mM Tris-HCL (pH 7.5), 50 μM ATP, 10 mM MgCL_2_, 25 mM NaCL and 1 mM DTT at 30°C for 5 minutes. The protein-bound beads were washed three times with lysis buffer, and then eluted with SDS sample buffer. The proteins were separated by SDS-PAGE using a 4% to 12% gradient Bis-Tris gel (Invitrogen), and stained with Silver Stain DAIICHI (Daiichi Pure Chemicals, Tokyo, Japan). An approximately 30 kDa band, which was seen in pulled-down cell lysates with WT-MELK added but not in those with D150A-MELK added, was extracted. Its peptide sequence was determined by MALDI-TOF mass spectrometry.

### Western blot analysis

To detect the endogenous MELK and Bcl-G_L _proteins in breast cancer cell lines (BT-20, HBC4, HBC5, HBL100, MCF-7, MDA-MB-231, SKBR3 and T47D) and HMECs, these cells were lysed in lysis buffer (50 mM Tris-HCL pH 8.0, 150 mM NaCL, 0.5% NP-40) including 0.1% protease inhibitor cocktail III (Calbiochem). After homogenization, the cell lysates were incubated on ice for 30 minutes and centrifuged at 14,000 rpm for 15 minutes to separate only supernatant from cell debris. The amount of total protein was estimated by protein assay kit (Bio-Rad), and the proteins were then mixed with SDS sample buffer and boiled for 3 minutes before loading into a 10% to 20% gradient SDS-PAGE gel (Bio-Rad). After electrophoresis, the proteins were transferred onto nitrocellulose membrane (GE Healthcare). Membranes were blocked by 4% BlockAce (Dainippon Pharmaceutical Co., Ltd, Osaka, Japan), and incubated with anti-MELK polyclonal or anti-Bcl-G_L _polyclonal antibodies (Abcam, Cambridge, MA, USA). β-Actin served as a loading control. Finally, the membranes were incubated with horseradish peroxidase (HRP) conjugated secondary antibody and protein bands were visualized by enhanced chemiluminescence (ECL) detection reagents (GE Healthcare).

### Co-immunoprecipitation assay

HeLa cells were transiently co-transfected for 48 hours with 8 μg of plasmid constructs encoding Flag-tagged full-length Bcl-G_L _or a series of Flag-tagged partial Bcl-G_L _proteins (FL, N-1, N-2, N-3, N-4, C-1, C-2 and C-3) as well as the same amount of plasmid encoding HA-tagged WT-MELK, using the FuGENE6 transfection reagent (Roche). Cells were lysed with lysis buffer as described above. The lysates were pre-cleaned with normal mouse IgG (1.2 μg) and rec-Protein G Sepharose 4B (Zymed, San Francisco, CA, USA) at 4°C for 30 minutes. Subsequently, the lysate was incubated with anti-Flag agarose M2 gel (Sigma-Aldrich) at 4°C for 12 hours. After washing three times with lysis buffer, proteins on beads were eluted with SDS sample buffer.

### *In vitro *binding assay

The His-tagged MELK recombinant protein (10 μg) was immunoprecipitated with anti-6XHis-tag monoclonal antibody (2 μg; Takara Clontech) in 500 μl of coupling buffer (50 mM Tris, 75 mM NaCL, 10 mM MgCL_2_, 1 mM DTT, and complete EDTA-free protease inhibitors; Roche) for 3 hours at 4°C. Subsequently, 30 μl of protein G Sepharose (Zymed) was added, and the incubation was further continued for 45 minutes. The immune complexes were washed 3 times with 1 ml of coupling buffer, and resuspended with 500 μl of binding buffer (50 mM Tris, 75 mM NaCL, 10 mM MgCL_2_, 1 mM DTT, 2% bovine serum albumin, 100 μM ATP and complete EDTA-free protease inhibitors; Roche) containing 5 μg of Bcl-G_L _recombinant protein. After incubation for 30 minutes at 4°C for binding, the samples were washed 5 times with washing buffer (50 mM Tris-CL, 75 mM NaCL, 10 mM MgCL_2_, 1 mM DTT, 0.01% Triton-X100 and complete EDTA-free protease inhibitors; Roche). The precipitates were then eluted with SDS sample buffer by boiling for 3 minutes and analyzed by western blot with anti-Bcl-G_L _antibody.

### Immunocomplex kinase assay

To use the long isoform Bcl-G_L _recombinant protein as a substrate for kinase assay, a set of Flag-tagged Bcl-G_L _(FL, N-1, N-2 N-3 and N-4) expression vectors was transfected into HeLa cells, and the proteins were immunoprecipitated with Flag-conjugated agarose M2 gel (Sigma-Aldrich) at 4°C for 1 hour. These immunoprecipitates were washed five times with lysis buffer containing 50 mM Tris-HCL (pH 7.5), 150 mM NaCL, 1% NP-40 and 0.1% protease inhibitor cocktail III (Calbiochem). Aliquots (20 μl) of immunoprecipitates were subjected to immunoblotting using rabbit anti-Flag antibody (Sigma-Aldrich) to check the success of the immunoprecipitation experiments. His-tagged MELK recombinant proteins (0.5 μM WT-MELK and D150A-MELK) were reacted for 30 minutes at 30°C with aliquots (20 μl) of immunoprecipitates of Flag-tagged Bcl-G_L _(FL, N-1 N-2, N-3 and N-4) as substrates in 50 μl of kinase buffer containing 30 mM Tris-HCL (pH 7.5), 0.1 mM EGTA, 10 mM DTT, 40 mM NaF, 40 mM sodium β-glycerophosphate (Sigma-Aldrich), 50 μM cold-ATP, 10 Ci of [γ-^32^P] ATP (GE Healthcare) and 10 mM MgCL_2_. The reaction was terminated by addition of SDS sample buffer and boiled for 3 minutes prior to 10% SDS-PAGE. The gel was then dried and autoradiographed with intensifying screens at room temperature overnight.

### TUNEL assay for apoptosis

COS7 cells (1 × 10^6^) were co-transfected with 8 μg each of Flag-tagged pCAGGSn3FH vectors (Flag-Bcl-G_L _and Flag-Mock) and HA-tagged pCAGGSnHC vectors (HA-WT-MELK, HA-D150A-MELK and HA-Mock) using the FuGENE 6 transfection reagent (Roche) according to the supplier's protocol. At 24 hours post-transfection, cells were harvested by trypsinization and washed with phosphate buffered saline without CaCL_2 _and MgCL_2 _(PBS(-)). An aliquot of cells (300 μl) was immobilized on the microslide glass (Matsunami Glass, Osaka, Japan) and subjected to a TUNEL assay using an *In situ *Cell Death Detection Kit, Fluorescein (Roche) according to the supplier's instructions. The apoptotic cells were observed with a TCS SP2 AOBS microscope (Leica, Tokyo, Japan). Experiments were carried out in triplicate independently.

### Flow cytometry analysis for apoptosis

COS7 cells (1 × 10^6^) were co-transfected with 8 μg each of Flag-tagged pCAGGSn3FH vectors (Flag-Bcl-G_L _and Flag-Mock) and HA-tagged pCAGGSnHC vectors (HA-WT-MELK, HA-D150A-MELK and HA-Mock) using the FuGENE 6 transfection reagent (Roche). For FACS analysis, the cells were harvested by trypsinization and fixed with 70% ethanol at room temperature for 30 minutes. After centrifuging to remove the ethanol, the cells were treated with 0.5 ml of PBS(-) containing 1 mg/ml of RNase I (Sigma-Aldrich) for 30 minutes followed by staining in 1 ml of PBS(-) containing 20 mg/ml of propidium iodide (Sigma-Aldrich) for 30 minutes. The cells selected from at least 10,000 ungated cells were analyzed for DNA content by flow cytometry (FACS calibur; Becton Dickinson, San Diego, CA, USA). The data were analyzed using CELLQuest software (FACS calibur; Becton Dickinson). Assays were done in triplicate independently.

### Statistical analysis

Statistical significance was determined by Student's *t*-test using Statview 5.0 software (SAS Institute, Cary, NC, USA). A difference of *P *< 0.05 was considered to be statistically significant.

## Results

### Up-regulation of *MELK *in breast cancer

**Figure 1:**
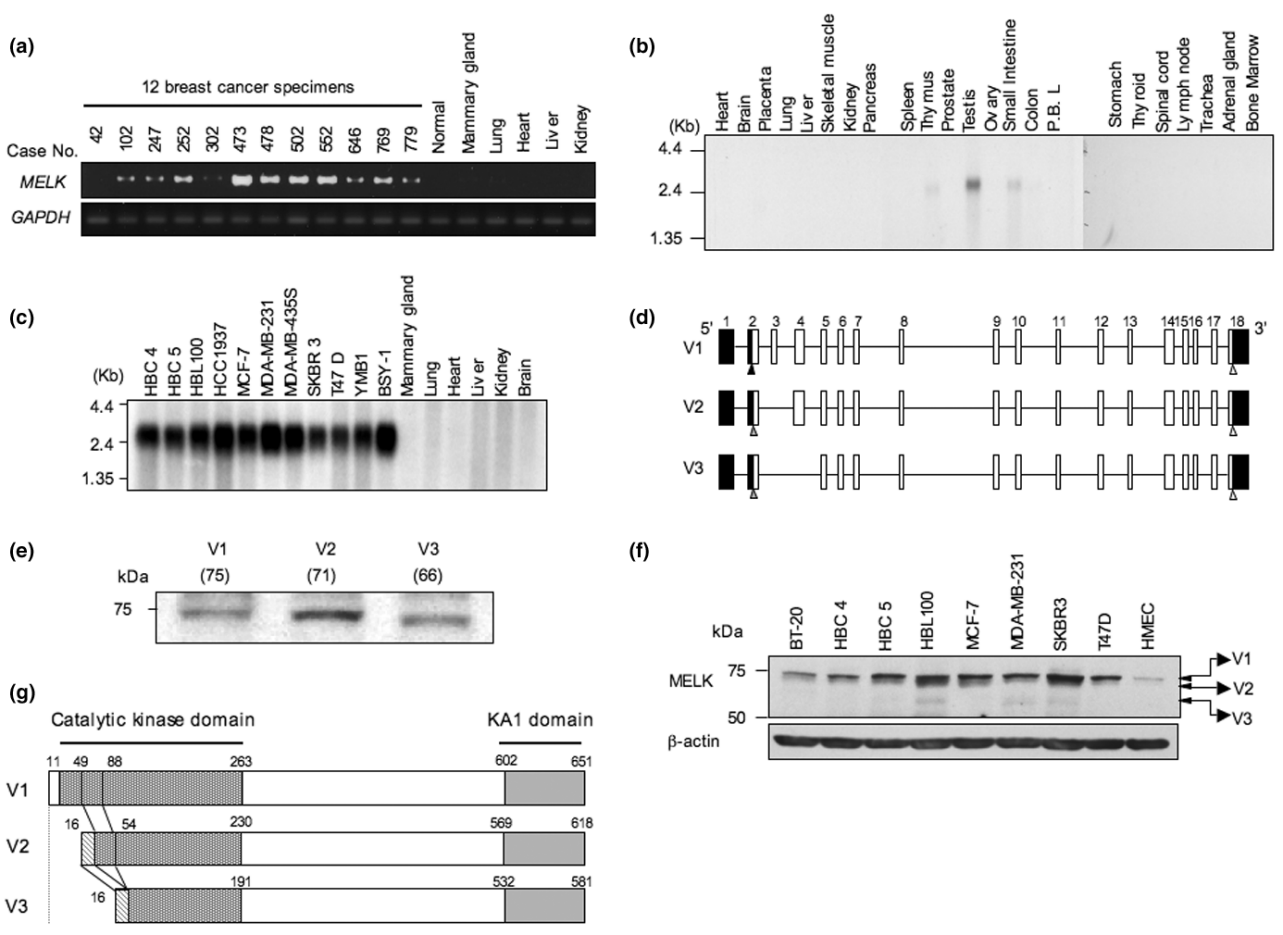
Expression and distribution of *MELK *in human normal tissues and breast cancer cell lines. **(a) **Expression of *MELK *in 12 breast cancer specimens (case number; 42, 102, 247, 252, 302, 473, 478, 502, 552, 646, 769 and 779) by semi-quantitative RT-PCR. *GAPDH *served as a quantitative internal control. **(b) **Multiple tissue Northern blot analysis demonstrated that an approximately 2.7 kb *MELK *transcript was detected in the testis, thymus and small intestine. PBL, peripheral blood leukocytes. **(c) **Breast cancer cell line Northern blot analysis revealed that approximately 2.4 to 2.7 kb *MELK *variants were specifically expressed in breast cancer cell lines, but not in normal vital organs. **(d) **Schematic representation of three variant transcripts identified by cDNA library screening (see Materials and methods). White boxes indicate a coding region and black boxes indicate a non-coding region. Black and grey triangles indicate initiation codons, and white triangles indicate stop codons. Exon numbers are shown above each box. **(e) ***In vitro *translation assay of each variant isolated from cDNA library screening. The number within parentheses represents the predicted molecular weight (kDa) of each variant protein. **(f) **Expression of MELK proteins in eight breast cancer cell lines as well as human mammary epithelial cells (HMECs) shown by western blot analysis with an anti-MELK antibody. β-Actin served as a control. **(g) **Schematic representation of the V1, V2 and V3 forms of MELK. The shaded boxes indicate the catalytic domain (amino acids 11 to 263 of the V1 protein). The KA1 domain is the kinase-associated domain in the carboxy-terminal region.

We previously reported the genome-wide expression profile analysis of 81 clinical breast cancers, representing 23,040 genes, by means of cDNA microarray analysis in combination with enrichment of cancer cells with a laser microbeam microdissection system [8]. The *MELK *gene (GenBank accession number NM_014791) was found to be one of the genes transactivated at a very high level in the great majority of the breast cancers examined. The subsequent semi-quantitative RT-PCR analysis of the transcript confirmed the elevated expression of *MELK *in 11 of 12 clinical breast cancer specimens (Figure 1a).

To further examine the expression pattern of this gene in breast cancer cell lines and normal tissues, we performed Northern blot analyses with mRNAs from multiple human tissues and breast cancer cell lines using a cDNA fragment corresponding to the 3' untranslated region of the *MELK *gene as a probe. The results showed that an approximately 2.7 kb band was most highly expressed in testis, and weakly expressed in thymus and small intestine (Figure 1b). Interestingly, Northern blot analyses of breast cancer cell lines using the same probe showed that 2.4 to 2.7 kb bands were significantly over-expressed in breast cancer cells but were not expressed in vital organs (Figure 1c), indicating that some cancer specific transcripts are present over this range.

To characterize the variants specifically transcribed in breast cancer cells, we screened a cDNA library of breast cancer cells. Subsequent cDNA sequencing analysis identified three different transcriptional variants, designated V1 (2,501 bp; NM_014791), V2 (2,368 bp; AB183427) and V3 (2,251 bp; AB183428). These three different transcriptional variants consist of 18, 17 and 16 exons, respectively. The V2 variant lacks exon 3 (86 bp) and the V3 variant lacks exon 3 (86 bp) and exon 4 (117 bp) as a result of alternative splicing (Figure 1d). The V2 and V3 variants contain early stop codons (TGA) in exon 4 and exon 5, respectively, if they are translated from the same first ATG codon of the V1 variant. However, if they are translated from a second ATG codon located downstream of the first ATG codon of the V1 variant, the V2 and V3 variant proteins are thought to produce 618 and 581 amino acid peptides, respectively. Thus, to examine both possibilities, we carried out an *in vitro *translation assay and found that the V1, V2 and V3 proteins were 75, 71 and 66 kDa, respectively, indicating that the V2 and V3 proteins were translated from the second ATG codon (Figure 1e).

Subsequently, we examined MELK expression in breast cancer cell lines by western blot analysis with anti-MELK polyclonal antibodies. The results indicate that the V1 protein is dominantly overexpressed in breast cancer cells compared with the V2 and V3 proteins (Figure 1f). Furthermore, although all of the three variant proteins possess the KA1 domain in their carboxyl terminus, the V2 and V3 proteins lack the amino-terminal portion, which corresponds to the first 48 amino acids of the V1 protein and is considered to be the catalytic kinase domain (Figure 1g). Although we can not rule out the possibility that the V2 and V3 variants have some functional role in carcinogenesis, for example, dominant-negative functions, in this study we focus on the kinase activity of the V1 transcript due to its predominant expression in cancer cells.

### Growth-inhibitory effects of siRNA against *MELK*

**Figure 2:**
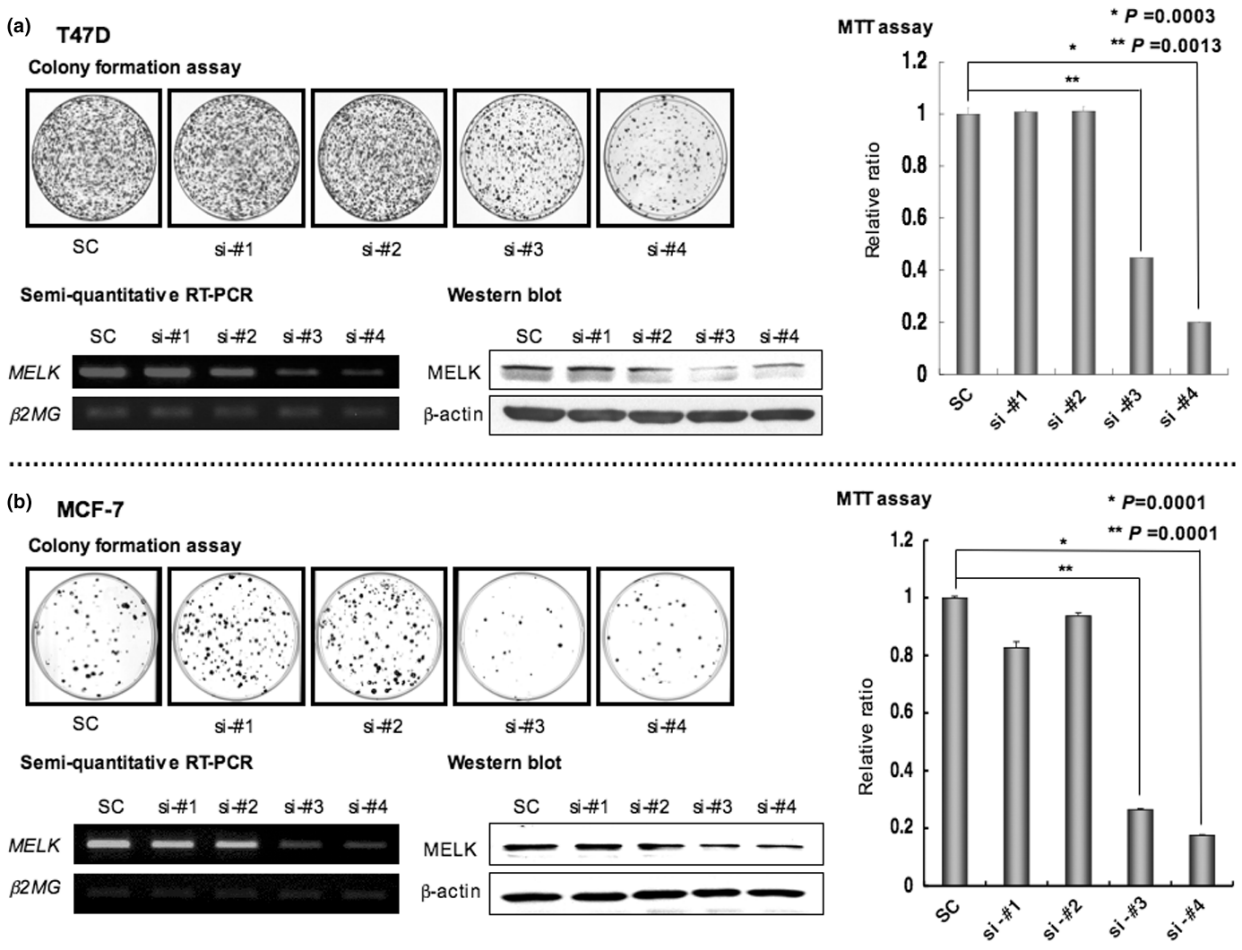
Effect of knockdown of *MELK *by small-interfering RNA (siRNA) on cell viability and proliferation. Four psiH1 promoter-based siRNA constructs (si-#1, si-#2, si-#3 and si-#4) were introduced into **(a) **T47D and **(b) **MCF-7 cell lines. SC refers to scramble used as a control for siRNA experiments. Gene silencing was evaluated by semi-quantitative RT-PCR and western blot analyses at four and five days after neomycin selection, respectively. β*2-microglobulin *(β*2MG*) was used as a control for normalization of semi-quantitative RT-PCR, and β-actin was used as a control in western blot analysis. MTT (3-(4,5-dimethylthiazol-2-yl)-2,5-diphenyltetrazolium bromide) assays were performed to evaluate cell viability at 10 days after neomycin selection, and graphed after standardization using the scramble control (SC) as 1.0 (T47D, *P *= 0.0003, *P *= 0.0013; MCF-7, *P *= 0.0001, *P *= 0.0001; unpaired *t*-test). Colony formation assays were carried out three weeks after neomycin selection (see Materials and methods). Two siRNA constructs (si-#3 and -#4) showed significant knockdown effects against internal *MELK *expression and inhibited cell growth in both T47D (a) and MCF-7 (b) cell lines. Values represent the average from triplicate experiments. Error bars indicate standard deviation.

To assess a possible role of *MELK *in mammary carcinogenesis, we knocked down the expression of endogenous *MELK *in the breast cancer cell lines T47D and MCF7 (Figure 2a,b), in which *MELK *was overexpressed at a high level, by means of the mammalian vector-based RNAi technique (see Materials and methods). We examined expression levels of *MELK *by semi-quantitative RT-PCR and western blot analyses, and found that the *MELK*-siRNAs si-#3 and si-#4 significantly reduced its expression at the transcriptional and protein levels compared with scramble-siRNA (SC), si-#1 and si-#2 (Figure 2). Colony formation and MTT assays revealed that both si-#3 and si-#4 significantlysuppressed growth of T47D and MCF7 cells (MTT assay in T47D, *P *= 0.0003, *P *= 0.0013; in MCF-7, *P *= 0.0001, *P *= 0.0001; unpaired *t*-test), in concordance with the results showing the knockdown effect of this gene. These results suggest that *MELK *has a critical role in the growth of breast cancer cells.

### Identification of Bcl-G_L _as a MELK-interacting protein

**Figure 3:**
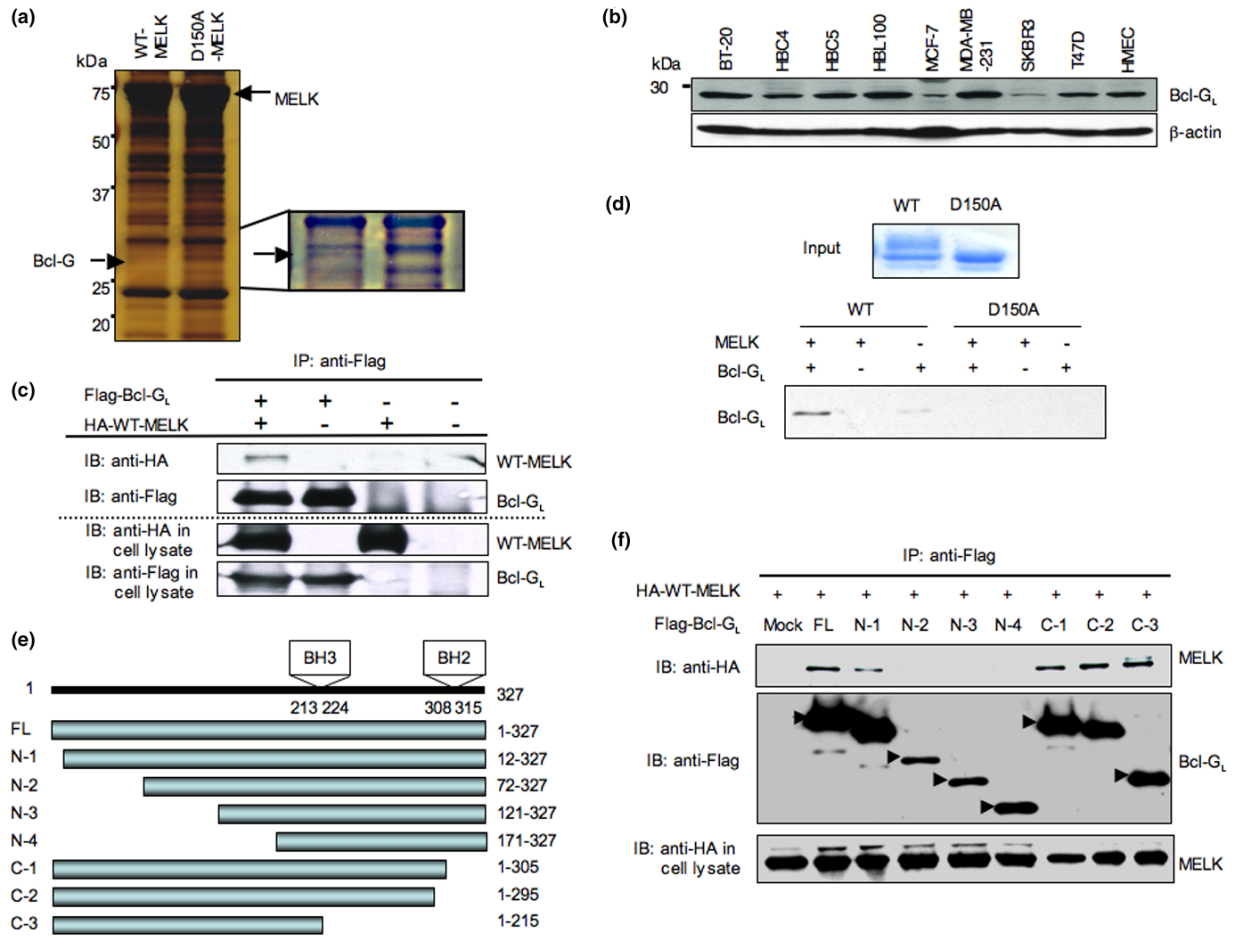
Identification of Bcl-G_L _as an interacting protein for MELK. **(a) **Silver staining of SDS-PAGE gels that contained the pulled-down cell lysates. The enlarged area covering Bcl-G shows the differential interaction between it and wild-type MELK (WT-MELK) and kinase-dead MELK (D150A-MELK). **(b) **Expression of Bcl-G_L _in eight breast cancer cell lines as well as human mammary epithelial cells (HMECs) by western blot analysis with an anti-Bcl-G_L _antibody. β-Actin was used as a control. **(c) **Interaction of MELK with Bcl-G_L_. Extracts from HeLa-cells transfected with HA (hemagglutinin)-tagged WT-MELK (HA-WT-MELK) or Flag-tagged Bcl-G_L _(Flag-Bcl-G_L_), or a combination of these, were harvested 36 hours after transfection. The cell lysates were immunoprecipitated with anti-Flag M2 antibody. Precipitated proteins were separated by SDS-PAGE and western blotting analysis was performed with an anti-HA antibody. **(d) **Direct interaction of the MELK and Bcl-G_L _proteins. The upper panel indicates the amount of input of WT-MELK and D150A-MELK. His-tagged WT-MELK bound to Bcl-G_L_, but His-tagged D150A-MELK did not. **(e) **Schematic representation of the amino- and carboxy-terminal deletion constructs of Bcl-G_L_. The C-1 and C-2 constructs have the BH2 domain deleted, and the C-3 construct has both the BH2 and BH3 domains deleted. **(f) **Determination of the WT-MELK binding regions of Bcl-G_L _by immunoprecipitation. The HA-tagged WT-MELK and various peptide sequences of Flag-tagged Bcl-G_L _(Figure 3e) were pulled down by immunoprecipitation with Flag-M2 antibody and then immunoblotted with rabbit anti-Flag antibody. The expression of HA-tagged WT-MELK in total cell lysates was confirmed by western blotting analysis. As a control, immunoprecipitation was performed from cells co-transfected with pCAGGSn3FC (Mock) and HA-tagged WT-MELK (HA-WT-MELK) through all steps. Arrowheads indicate expression of each Bcl-G_L _peptide.

To investigate the biological functions of MELK in breast cancer cells, we searched for substrates of MELK in cancer cells by *in vitro *protein pull-down assays using wild-type MELK (WT-MELK) and kinase-dead MELK (D150A-MELK) recombinant proteins. Comparison of silver staining of SDS-PAGE gels containing the pulled-down proteins identified an approximately 30 kDa protein in the lane corresponding to proteins pulled-down with WT-MELK but not in that corresponding to proteins pulled-down with D150A-MELK (Figure 3a). MALDI-TOF analysis showed this 30 kDa protein to be Bcl-G, a member of the Bcl-2 protein family [14]. According to NCBI database, *Bcl-G *has three alternative splicing isoforms, termed *Bcl-G *short isoform (*Bcl-G*_*S*_), median isoform (*Bcl-G*_*m*_) and long isoform (*Bcl-G*_*L*_), and encoding 252, 276 and 327 amino acid peptides, respectively.

We first examined the expression patterns of these *Bcl-G *isoforms at the transcriptional level in breast cancer cell lines by semi-quantitative RT-PCR, and found that *Bcl-G*_*S *_and *Bcl-G*_*L *_were expressed in all breast cancer cells examined, whereas the *Bcl-G*_*m *_transcript was not detected at all (data not shown). Furthermore, we examined expression of endogenous Bcl-G_L _in breast cancer cell lines at the protein level by western blot analysis using an anti-Bcl-G_L _antibody and detected an approximately 30 kDa endogenous Bcl-G_L_, corresponding to the size identified by protein pull-down experiment, in all the breast cancer cell lines examined (Figure 3b). Therefore, we focused on Bcl-G_L _as a candidate interacting protein for MELK.

To validate an interaction between WT-MELK and Bcl-G_L_, we constructed plasmids designed to express HA-tagged WT-MELK (HA-WT-MELK) and Flag-tagged Bcl-G_L _(Flag-Bcl-G_L_). These plasmids were co-transfected into HeLa cells and the proteins immunoprecipitated with anti-Flag antibody. Immunoblotting of the precipitates using anti-HA antibodies indicated that Flag-Bcl-G_L _was co-precipitated with HA-WT-MELK (Figure 3c). Furthermore, we demonstrated that His-tagged WT-MELK could pull-down with Bcl-G_L _but His-tagged D150A-MELK could not, indicating that, *in vitro*, Bcl-G_L _interacts directly with WT-MELK but not with D150A-MELK (Figure 3d). To further determine which segment of Bcl-G_L _can interact with WT-MELK, we performed co-immunoprecipitation analyses using HA-WT-MELK and partial Bcl-G_L _proteins tagged with Flag (Figure 3e). After co-transfection of plasmid clones into HeLa cells, we performed immunoprecipitation with an anti-Flag antibody, and then immunoblotting with an anti-HA antibody. We found that the N-1 construct (amino acids 12 to 327) as well as a full-length Bcl-G_L _(FL) bound to WT-MELK, whereas N-2 (amino acids 72 to 324), N-3 (amino acids 121 to 327) or N-4 (amino acids 171 to 327) did not (Figure 3f). Concordantly, all of the partial products that contained the amino-terminal portion of Bcl-G_L _– C-1 (amino acids 1 to 305), C-2 (amino acids 1 to 295), and C-3 (amino acids 1 to 215) bound to WT-MELK (Figure 3f). These results suggest that the region corresponding to amino acids 12 to 71 of Bcl-G_L _is likely to interact with WT-MELK.

### MELK phosphorylates Bcl-G_L_

**Figure 4:**
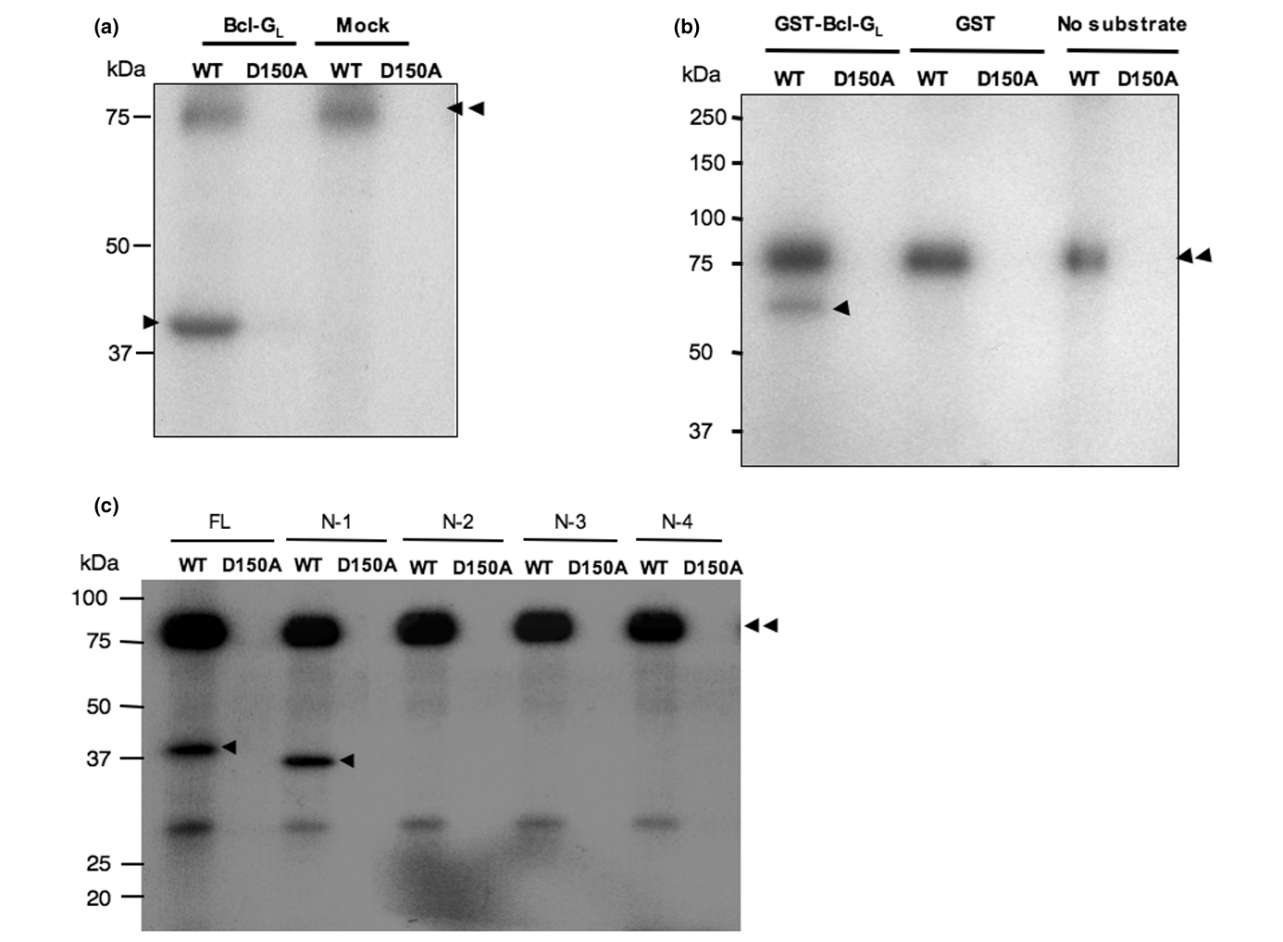
MELK phosphorylates Bcl-G_L _*in vitro*. **(a) **Immunoprecipitates were subjected to immune complex kinase assay with wild-type (WT)-MELK or kinase-dead (D150A)-MELK). The single arrowhead indicates phosphorylated Bcl-G_L_, and the double arrowhead points to an autophosphorylated MELK protein. **(b) **Phosphorylation of a bacterial glutathione S-transferase (GST) Bcl-G_L _fusion recombinant protein (GST-Bcl-G_L_) by His-tagged WT-MELK (WT). The single arrowhead indicates phosphorylated GST-Bcl-G_L _protein, and the double arrowhead indicates an autophosphorylated His-tagged MELK protein. **(c) ***In vitro *phosphorylation of various partial amino-terminal constructs of Bcl-G_L _(N-1, N-2, N-3 and N-4; see Figure 3e) and full-length Bcl-G_L _(FL) by MELK. The single arrowheads indicate phosphorylated immunoprecipitated Bcl-G_L _proteins, and the double arrowhead indicates an autophosphorylated MELK recombinant protein.

To examine whether the Bcl-G_L _protein is a substrate of MELK kinase activity, we performed an immune complex kinase assay. We first confirmed the exogenous expression of Flag-tagged Bcl-G_L _(Flag-Bcl-G_L_) by western blot analysis (Figure 3f). As shown in Figure 4a, WT-MELK could phosphorylate Bcl-G_L_, but D150A-MELK could not (single arrowhead). In addition, autophosphorylation of an approximately 75 kDa protein of MELK was observed (Figure 4a; double arrowheads). Furthermore, we also confirmed that WT-MELK could phosphorylate GST-Bcl-G_L _but D150A-MELK could not (Figure 4b), indicating MELK can directly phosphorylate Bcl-G_L_. These findings suggest that Bcl-G_L _is a potential substrate for MELK.

We subsequently examined WT-MELK kinase activity on various partial amino-terminal Bcl-G_L _products (Figure 3e). First, we confirmed the amount of immunoprecipitated protein corresponding to partial Bcl-G_L _(N-1, N-2, N-3 and N-4) as well as to full-length Bcl-G_L _(FL) by western blot analysis (Figure 3f), and then examined the extent of their phosphorylation (Figure 4c). The MELK recombinant protein phosphorylated N-1 as well as full-length Bcl-G_L_, whereas it could not phosphorylate N-2, N-3 or N-4 proteins, corresponding with their inability to interact.

### MELK involvement in the apoptotic pathway through Bcl-G_L_

**Figure 5:**
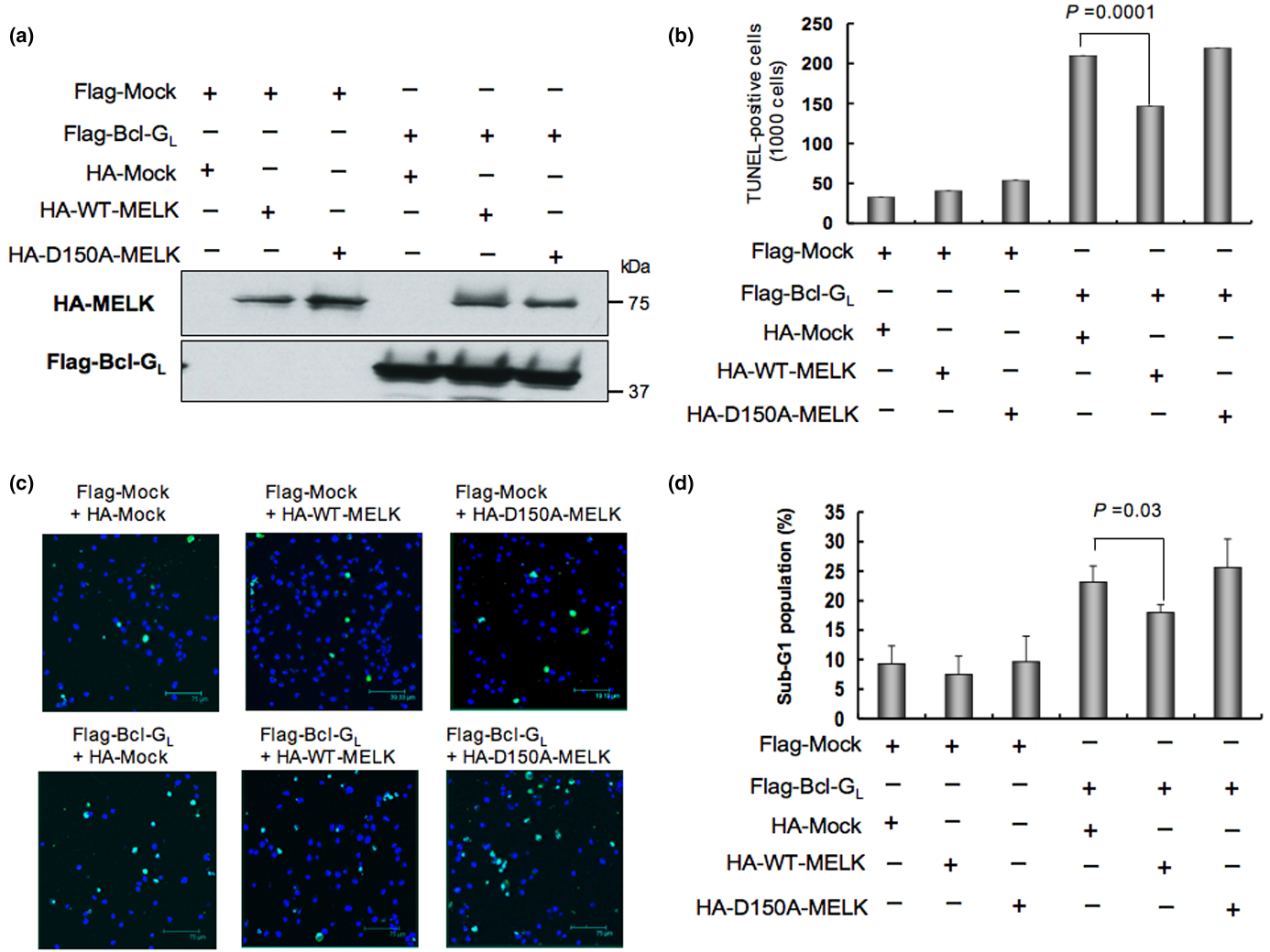
MELK involvement in the apoptosis cascade through Bcl-G_L_. HA (hemagglutinin)-tagged MELK (HA-wild-type (WT)-MELK or HA-kinase-dead (D150A)-MELK) and Flag-tagged Bcl-G_L _(Flag-Bcl-G_L_) expression vectors were co-transfected into COS7 cells for 24 hours. **(a) **The expression of MELK and Bcl-G_L _proteins in the co-transfected cells were examined by western blot analysis. **(b) **TUNEL assays after transfection with pCAGGSnHC (HA-Mock), pCAGGSn3FH (Flag-Mock), HA-tagged MELK (WT and D150A), Flag-tagged Bcl-G_L _expression vectors, and combinations of these. Apoptotic cells were measured by counting of TUNEL staining (means ± standard deviation, *n *= 3; *P *= 0.0001; unpaired *t*-test). **(c) **Representative images of TUNEL assays. Cells were labeled with DAPI (4',6-diamidino-2-phenylindole) for counting of total cell number. Apoptotic cells with DNA strand breaks were labeled with green fluorescence. **(d) **FACS analysis of cells collected after transfection with pCAGGSnHC (HA-Mock), pCAGGSn3FH (Flag-Mock), HA-tagged MELK (WT and D150A), Flag-tagged Bcl-G_L _expression vectors, and combinations of these. Proportions of apoptotic cells are indicated as percentages of sub-G1 populations. Each value represents the average of three experiments (means ± standard deviation, *n *= 3).

Because MELK can physically interact with and phosphorylate Bcl-G_L _(Figures 3d and 4a), we hypothesized that it might be involved in the apoptosis cascade through the Bcl-G_L_-related pathway. To investigate this hypothesis, we transiently co-transfected two plasmid clones designed to express HA-tagged MELK (WT or D150A) and Flag-tagged Bcl-G_L _into COS7 cells, and then performed a TUNEL assay and FACS analysis to measure the proportions of apoptotic cells (see Material and methods). We first confirmed the exogenous expression of MELK and Bcl-G_L _in COS7 cells by western blot analysis (Figure 5a). As indicated in Figure 5b,c, the overexpression of full-length Bcl-G_L _(HA-Mock+Flag-Bcl-G_L_) significantly increased the proportion of TUNEL-positive cells compared with the cells transfected with the mock plasmids (HA-Mock+Flag-Mock), indicating that Bcl-G_L _induces apoptosis, as described previously [14]. In contrast, the co-overexpression of WT-MELK with Bcl-G_L _(HA-WT-MELK+Flag-Bcl-G_L_) reduced the proportion of TUNEL-positive cells compared with over-expression of Bcl-G_L _alone (HA-Mock+Flag-Bcl-G_L_) (*P *= 0.0001, unpaired *t*-test). However, the co-overexpression of D150A-MELK with Bcl-G_L _(HA-D150A-MELK+Flag-Bcl-G_L_) did not affect the proportion of TUNEL-positive cells. As shown in Figure 5d, FACS analysis of the cells under the same conditions also confirmed that the overexpression of Bcl-G_L _increased the sub-G1 population of cells compared with the mock-transfected cells. Similarly to the TUNEL analysis, the overexpression of WT-MELK with Bcl-G_L _reduced the proportion of sub-G1 cells (*P *= 0.03, unpaired *t*-test), while D150A-MELK increased the sub-G1 population. Our results imply that the kinase activity of MELK may play a critical role in the regulation of the pro-apoptotic function of Bcl-G_L_.

## Discussion

Microarray technologies applied to the study of cancer have contributed to the discovery of genes that play essential roles in carcinogenesis, the discovery of novel molecular target(s) for the development of anti-cancer agents and/or diagnosis, and the identification of genes related to chemo- or radio-sensitivity. Although such information has been applied in the development of novel therapeutic agents for breast cancer, the range of available treatments for advanced-stage patients is still very limited. Thus, it is urgent that further molecular targets are discovered to enable development of new anti-cancer drugs that are specific for malignant cells with a minimum risk of adverse reactions.

Using genome-wide expression profiles of breast cancers and normal human tissues [8,9], we identified *MELK*, one of the transcripts of which was specifically up-regulated in the great majority of clinical breast cancer samples, but expressed in none of 29 normal human tissues examined except testis, thymus and small intestine.

In this study, we characterize the biological function of MELK and indicate that it would be a good candidate as a molecular target for breast cancer therapy, although there would be some concerns over intestinal adverse reactions. We demonstrated by means of siRNA that knockdown of endogenous MELK expression results in growth suppression of breast cancer cells (Figure 2). Furthermore, our cDNA microarray data indicate that *MELK *is also up-regulated relatively frequently in bladder cancers, osteosarcoma and small cell lung cancers (data not shown). Together, these findings suggest that *MELK *has an oncogenic role in not only breast cancers but also bladder cancers, bone cancers and small cell lung cancers.

*MELK *was previously identified as a new member of the snf1/AMPK serine-threonine kinase family that is involved in mammalian embryonic development [15-18]. This gene was shown to play an important role in hematopoiesis, stem cell renewal [19] and cell-cycle progression through an interaction with zinc finger-like protein ZPR9 [20] and splicing factor NIPP1 [12]. To investigate the expression pattern of these proteins in breast cancer cells, we performed semi-quantitative RT-PCR, and observed no significant correlation of gene expression between *MELK *and these interacting molecules in breast cancer cells (data not shown). Thus, to investigate the biological significance of MELK in breast cancer cells, we searched for a possible substrate(s) of MELK by means of *in vitro *pull-down assays with recombinant wild-type MELK (WT-MELK) and kinase-dead MELK (D150A-MELK). We identified a long isoform of Bcl-G (Bcl-G_L_), a pro-apoptotic member of the Bcl-2 family, as a potential substrate for MELK. In addition to physical interaction between these two proteins, *in vitro *immune complex kinase and *in vitro *binding assays showed that Bcl-G_L _was directly bound to MELK and specifically phosphorylated by it *in vitro *(Figures 3d and 4a,b).

As reported previously [14], we demonstrated by TUNEL assay and FACS analysis that introduction of full-length Bcl-G_L _into COS7 cells induced apoptosis. However, under the same conditions, addition of exogenous WT-MELK suppressed induction of apoptosis by Bcl-G_L_, but addition of D150A-MELK did not (Figure 5b–d). Thus, we speculate that MELK might promote cell growth by inhibiting the pro-apoptotic function of Bcl-G_L _through its phosphorylation.

## Conclusion

Our findings clearly suggest that *MELK *is overexpressed in both breast cancer specimens and cancer cell lines, and that its kinase activity possibly plays a significant role in breast cancer cell growth. Recent anti-cancer drug development is focused on targeting important molecules involved in the oncogenic pathways, represented by imatinib mesylate and trastuzumab. We found that down-regulation of *MELK *by treatment with siRNA significantly suppressed the cell growth of breast cancer, indicating its crucial role in the proliferation and tumorgenesis of breast cancer. In particular, we demonstrate a new biological function for *MELK *in breast carcinogenesis, the inhibition of apoptosis though its interaction with and phosphorylation of Bcl-G_L_, a pro-apoptotic member of Bcl-2 family. Our data should contribute to a better understanding of breast carcinogenesis, and they suggest that *MELK *is a promising molecular target for breast cancer treatment.

## Abbreviations

β2MG = beta-2-microglobulin; DTT = dithiothreitol; GST = glutathione S-transferase; Her-2/ErbB-2 = epidermal growth factor 2; HMEC = human mammary epithelial cell; MTT = 3-(4,5-dimethylthiazol-2-yl)-2,5-diphenyltetrazolium bromide; PBS = phosphate-buffered saline; RNAi = RNA interference; SC = scramble control.

## Competing interests

The authors declare that they have no competing interests.

## Authors' contributions

LML performed all experiments and drafted the manuscript. PJH participated in the discussion and interpretation of data. NT constructed expression profiles of breast cancer and participated in the screening of candidate genes. YN was involved in the conception and design of studies, interpretation of data and preparing the final version of the manuscript. TK guided all molecular aspects of the studies and was involved in the design of studies and interpretation of results.
